# Anti-Corrosion Property of Glass Flake Reinforced Chemically Bonded Phosphate Ceramic Coatings

**DOI:** 10.3390/ma12132082

**Published:** 2019-06-28

**Authors:** Ge Yan, Mingyang Wang, Tao Sun, Xinping Li, Guiming Wang, Weisong Yin

**Affiliations:** 1 School of Civil Engineering and Architecture, Wuhan University of Technology, Wuhan 430070, China; 2 State Key Laboratory for Disaster Prevention & Mitigation of Explosion & Impact, Army Engineering University, Nanjing 210007, China; 3 State Key Laboratory of Silicate Materials for Architectures, Wuhan University of Technology, Wuhan 430070, China

**Keywords:** anti-corrosion property, glass flake, chemically bonded phosphate ceramic (CBPC), corrosion mechanism

## Abstract

Glass flake (GF) was used as the reinforcement in chemically bonded phosphate ceramic (CBPC) coatings to promote anti-corrosion property. The crystalline phase, curing behavior, micromorphology and electrochemical performance of the coatings were studied. The results indicate that with the addition of magnesia (MgO), a new bonding phase (Mg_3_(PO_4_)_2_) can be formed, which can help the CBPCs achieve a more compact and denser structure. The effect of the magnesia and the GF additives on curing behavior is obvious: the heat of reaction of the phosphate ceramic materials increases with the addition of the magnesia and the GF, which emphasizes the higher crosslinking density in the phosphate ceramic materials. The phosphate ceramic coatings with the magnesia have a higher impedance value compared with the neat phosphate ceramic coating, while the highest impedance value is obtained with increased content of GF. The corrosion mechanism is mainly contributed by the new bonding phase and GF particles, which can hinder the permeation pathway and make the permeation more circuitous.

## 1. Introduction

Reinforced concrete (RC) is one of the most commonly used modern construction materials. The corrosion of the interface between concrete and steel rebar affects the service life of RC seriously. In the past decades, in order to increase the lifespan of RC, researchers have dedicated efforts towards the enhancement of bond strength and corrosion resistance in RC [1,2]. Among all the anti-corrosion technology, a most effective way to improve the property of corrosion resistance is to cover the rebar with a durable and adhesive coating [3], which can be divided into organic coatings and inorganic coatings. Epoxy [4,5] and modified resin [6] have been generally adopted as organic coatings, while inorganic coatings include galvanizing metal coatings [7], chemically reactive enamel (CRE) coatings [8], and chemically bonded phosphate ceramic (CBPC) coatings [9]. In the application of the epoxy coating in reinforced concrete, Brown MC [10] arrived at the conclusion that the durability of the steel with the epoxy coating was not ideal due to the moisture that penetrates beneath the coating, which means that the epoxy-coated steel has a higher corrosion rate than bare steel. What’s more, the bone strength loss between the epoxy-coated steel and the concrete reached 20% compared to bare rebar [11]. For the inorganic coatings, a weakening effect on the bond strength between the galvanized reinforcing steel and the concrete was observed due to hydrogen evolution at the interface [12]. A high temperature of 800 °C needs to be applied to the chemically reactive enamel-coated steel, which can lead to a reduction in the creep limit and strength [1].

In recent years, more and more studies have focused on the utilization of CBPCs, which are derived from the reactions between base and acid, such as the metal oxide (Al_2_O_3_, MgO) and the soluble acid phosphate (KH_2_PO_4_, Al(H_2_PO_4_)_3_) [13,14]. Due to their corrosion resistance, mechanical resistance, thermal conductivity and low temperature used in their processing, CBPC coatings have considerable technological importance [15]. CBPC coatings can be seen to use the phosphate matrixes as the binding phases and the metal oxides as suitable fillers. Researchers [16,17,18] have investigated the influence of ceramic oxides (AlN, MgO, and ZrO_2_) on the improvement of the thermal properties of CBPC coatings. The abrasive filler of Al_2_O_3_-SiC in aluminum phosphate has been produced to enhance the abrasion resistance of coatings [19]. The ceramic coatings of aluminum phosphate formed by the reaction between soluble acid phosphates and alumina and alumina–sol–gel systems was studied [20]. H.M. Hawthorne et al. [21] prepared phosphate ceramic coatings on stainless steel substrates and analyzed the mechanical performance and electrochemical property. The application by thermal spraying for binders was carried out, and the microstructure defects of the coatings were improved [22]. A series of CBPC coatings were applied to steel surfaces to reduce or eliminate corrosion [23]. Da Bian et al. [24] paid attention to graphene-reinforced CBPC coatings. Zhu Ding et al. [25] studied the mechanical characterization and microstructure of CBPC composites reinforced with fiber, prepared at room temperature.

Glass flake (GF) particles are inorganic platelets with excellent resistance to chemicals and aging. Many efforts have been focused towards corrosion-resistant coatings with the advanced barrier properties of GF [26,27,28,29,30], and the parallel and overlapped arrangement of GF particles could form a compact impermeable layer in organic coatings. There are microstructure defects between GF particles and the organic coating matrix due to the coating matrix belonging to organic material, while GF belongs to inorganic materials; thus, we can only effectively improve the permeability resistance of organic coatings by surface treatment and functionalization of GF particles.

In all cases cited, there have been few reports on the application of GF particles as fillers in CBPC coatings. Meanwhile, the anticorrosion mechanism of the GF-reinforced CBPC coatings also needs to be illustrated. In this study, CBPC coatings reinforced with GF particles were prepared in order to protect the round steel. The crystalline phase, curing behavior, and micromorphology of the CBPC-based ceramic materials were analyzed, and the electrochemical characterization of the CBPC coatings was carried out in 3.5 wt.% NaCl solution using electrochemical measurement. Furthermore, the anticorrosion mechanism of the coating was also investigated.

## 2. Materials and Methods

### 2.1. Materials

The CBPC coatings reinforced with GF were applied onto round steel 8 mm in diameter and 20 mm in length by mixing raw materials of phosphate ceramic materials as shown in Table 1.

The preparation of monoaluminium phosphate (MAP) binder is based on the reaction between aluminum hydroxide and phosphoric acid at 120 °C under constant stirring for 60 min. The quantities were calculated according to Equation (1). For the preparation of MAP binder, 135.1 g of pure water were used to dilute 345.9 g of 85% phosphoric acid to 60%, and then 78.0 g of aluminum hydroxide were added.

3*H*_3_*PO*_4_ + *Al*(*OH*)_3_ → *Al*(*H*_2_*PO*_4_)_3_ + 3*H*_2_*O* (1)

**Figure 1:**
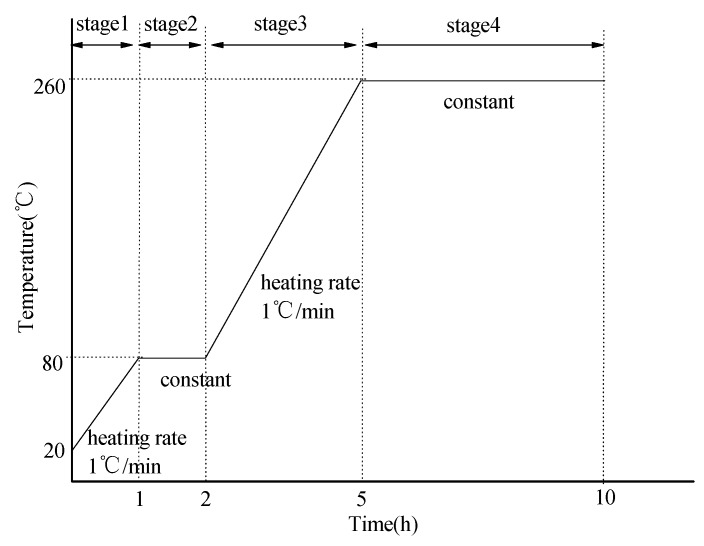
Curing process of phosphate ceramic coatings.

The mixture proportion of phosphate ceramic coating materials are shown in Table 2. The coated samples were prepared by brush coating with a clean bristle brush. Before preparing coating, 800 grit silicon carbide abrasive papers were used to polish the surface of the round steel, the round steel was degreased in acetone for 15 min (in ultrasonic bath) and then rinsed with deionized water. The coated round steel was placed at room temperature (25 ± 1 °C) for 10 h and then heated in an electric oven according to the curing process as shown in Figure 1.

### 2.2. Characterization

Laser scanning confocal microscopy (LSCM, manufacture, Oberkochen, Germany) was used to characterize the thickness and surface topography of GF. The thickness of the coating was tested using a QNIX4500 coating thickness gauge measurement (QNIX, Oberkochen, Germany) at a precision of 1 μm. The crystalline phases of phosphate ceramic coatings after curing were measured by X-ray diffractometer (PANalytical Empyrean, Almelo, The Netherlands) using a CuKα source scanning from 5° to 70° in 2θ. A differential scanning calorimeter (STA instruments, Selb, Germany) was used to analyze the curing behavior of phosphate ceramic coating materials under N_2_ atmosphere with gas flow of 30 mL/min, heat velocity of 10 °C/min, start temperature 25 °C and end temperature 400 °C. Furthermore, the SEM micrographs of the surface and cross-section of the coated samples were investigated on a JSM-IT300 (JEOL, Tokyo, Japan) under secondary electron mode, test voltage of 10 kV and surface treatment with platinum.

The potentiodynamic polarization was conducted using a workstation CS350 (Wuhan Corrtest Instrument Co., Ltd., Wuhan, China), as well as the electrochemical impedance spectroscopy (EIS) measurement, with a three-electrode system, containing the reference electrode (saturated calomel electrode), the counter electrode (platinum electrode), and the working electrode (sample). The electrochemical experiment was conducted in 3.5 wt.% NaCl solution at 25 ± 1 °C. The exposed surface area was around 2.5 cm^2^. After the samples were immersed in the NaCl solution for 10 h, the potentiodynamic polarization was performed at a speed of 2 mV·s^−1^, from −100 mV to 100mV. EIS measurements were carried out with AC signals of 5 mV peak-to-peak amplitude in the frequency start at 100 kHz and end at 0.01 Hz. Z-View software was used to evaluate the EIS data. At least three repeated electrochemical tests were carried out to confirm the reliability of the measurement.

## 3. Results and Discussions

### 3.1. Characterization of GF

**Figure 2:**
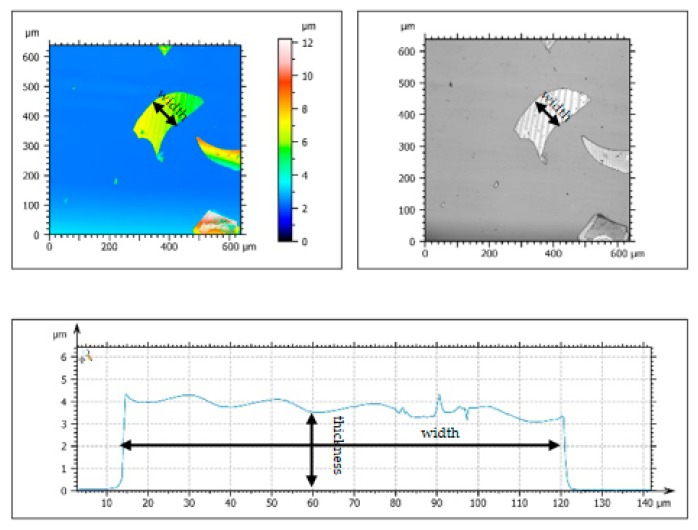
Surface topography of GF.

**Figure 3:**
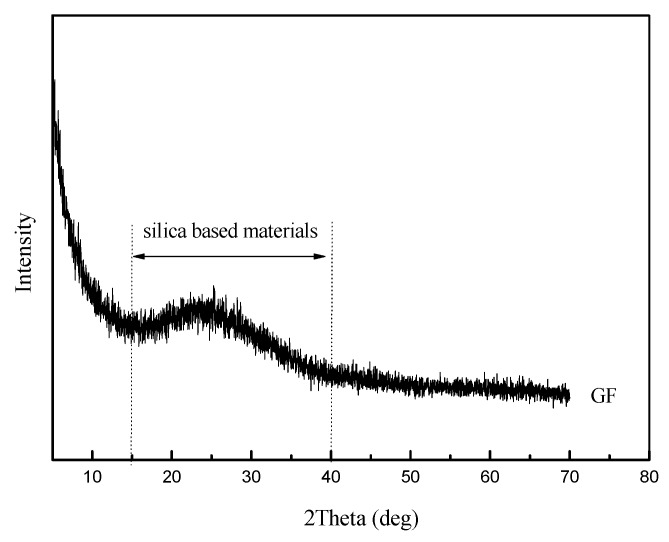
XRD patterns of GF.

As shown in Figure 2, the GF, with 150 mesh, has an average thickness of 3–4 µm and an average particle size of 105 µm. Meanwhile, the GF is irregularly formed and has a smooth surface topography. The amorphous peaks of GF in the XRD patterns were observed in the range of 15–40°, as can be seen in Figure 3, which means the GF particles are silica-based materials with a wide typical diffraction peak [31]. 

### 3.2. Characterization of Composites and Coatings

#### 3.2.1. Thickness of the Coatings

The thickness of the CBPC coatings is shown in Table 3. It is clear that the thickness of the different GCBPC coatings varied from 193 μm to 217 μm with the increase of GF particles, while the thickness of the CBPC coating was 186 μm. The coating thicknesses showed a little bit of increase, with regard to the standard deviation of the thickness measurement. The difference in thickness is not enough to affect the corrosion resistance of the coatings.

#### 3.2.2. XRD

**Figure 4:**
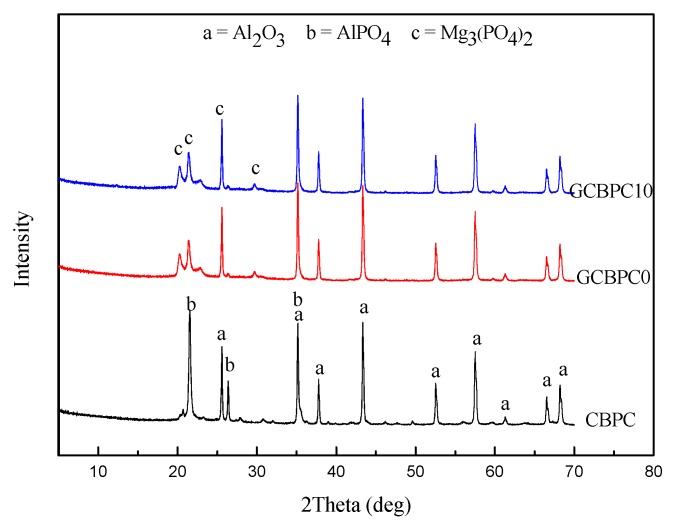
XRD patterns of phosphate ceramic coatings.

The results obtained from the XRD patterns of the phosphate ceramic coatings are shown in Figure 4. In the case of the CBPC, only Al_2_O_3_ and AlPO_4_ phase were present. While the unreacted Al_2_O_3_, MgO phase and the reacted AlPO_4_, Mg_3_(PO_4_)_2_ phase was identified in GCBPCs. A new bonding phase (Mg_3_(PO_4_)_2_) can be found, which may benefit the structural and anti-corrosion properties of the phosphate ceramic coatings, as it has fine needle-like crystals [15]. The new bonding phase was the reaction product of magnesia and MAP solution. MgO can fully react with MAP solutions in the current pastes, so only the product of Mg_3_(PO_4_)_2_ and its XRD peaks remain in the GCBPCs. The main binding phase in the phosphate ceramic coatings originates in these reactions of Equations (2) and (3) [15,32]. The reason for the higher peak of Mg_3_(PO_4_)_2_ in GCBPCs is related to two aspects. Firstly, the product of CBPC materials is largely limited by the solubility of metal oxides, which means the dissolved magnesia can react faster with MAP due to the higher solubility of the magnesia [33]. Secondly, the peak of AlPO_4_ phase may not be prominent in the presence of magnesia due to the covering of the Mg_3_(PO_4_)_2_ [34]. What’ s more, the results indicate that the phase of the GF did not alter under the heat treatment of the GCBPCs, which did not affect the crystalline phase of others.

(2) $$Al_{2}O_{3}+Al{\left(H_{2}PO_{4}\right)}_{3}\to 3AlPO_{4}+3H_{2}O$$

(3) $$3MgO+Al{\left(H_{2}PO_{4}\right)}_{3}\to AlPO_{4}+Mg_{3}{\left(PO_{4}\right)}_{2}+3H_{2}O$$

#### 3.2.3. Thermal Characterization

**Figure 5:**
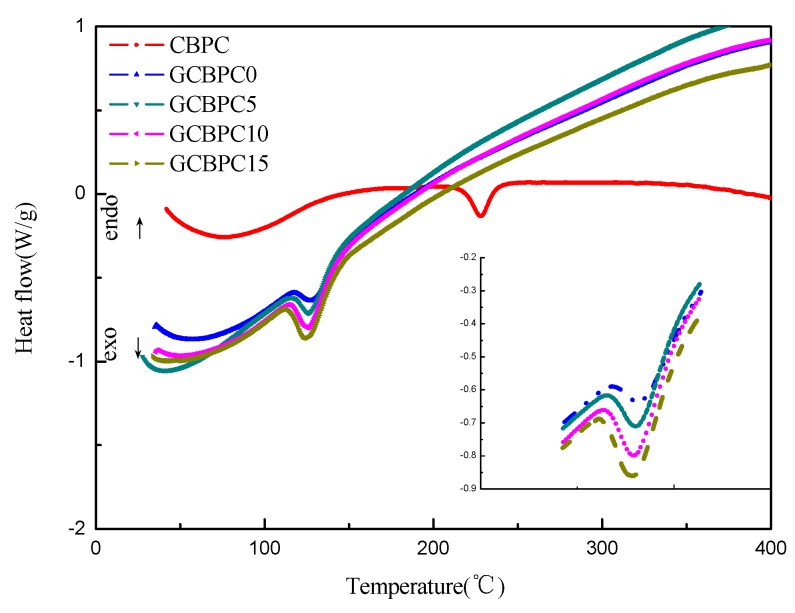
DSC curves of phosphate ceramic materials.

To evaluate the effects of the magnesia and the GF additives in the phosphate ceramic materials on the curing behavior, the DSC analysis of the phosphate ceramic materials before curing was conducted, which are shown in Figure 5. The temperature values of the onset (T_onset_), the peak (T_p_), the endset (T_endset_) and the curing enthalpy (ΔH) are available from the DSC curves, which are predicted in Table 4. All phosphate ceramic materials have only one significant endothermic peak. With the addition of magnesia, the endothermic peak temperature decreased from 228.0 °C (CBPC) to 126.9 °C (GCBPC0), which approaches the temperature of the maximum dissolution of the bauxite and magnesite [12]. Meanwhile, the endothermic peaks of coatings reinforced with GF are 126.3 °C (GCBPC5), 125.4 °C (GCBPC10), 125.2 °C (GCBPC15), respectively. 

The great changes in curing temperature can be seen clearly with the addition of magnesia [17]. The exothermic peak temperature (T_p_) of CBPC is 228 °C, while the GCBPCs are all around 125 °C. Besides, the ΔH are −30.32, −37.65, −48.30, −54.38, −56.39 J/g, respectively. Therefore, the curing enthalpy of reaction of the phosphate ceramic materials increases with the addition of the magnesia and the GF additives, which emphasizes the higher crosslinking density in the phosphate ceramic materials [35,36].

#### 3.2.4. SEM

**Figure 6:**
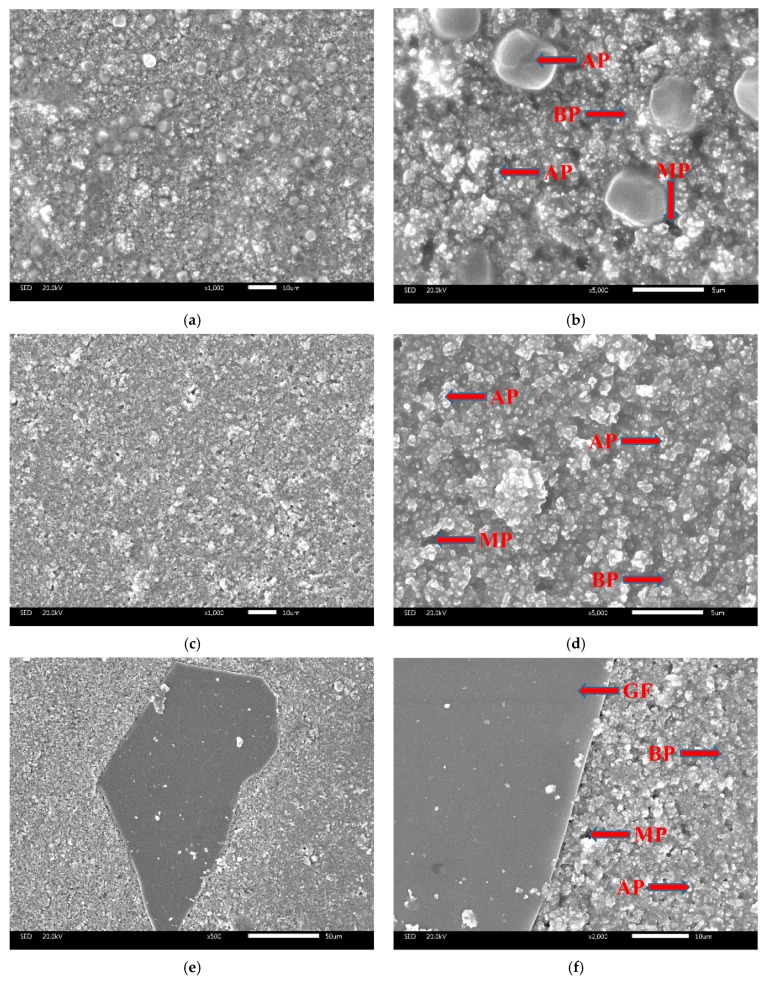
SEM micrographs of coating surfaces (after curing): (**a**) 1000× CBPC; (**b**) 5000× CBPC; (**c**) 1000× GCBPC0; (**d**) 5000× GCBPC0; (**e**) 500× GCBPC10; (**f**) 2000× GCBPC10. Notes: AP (alumina particles), BP (binding phases), MP (micro-porous).

**Figure 7:**
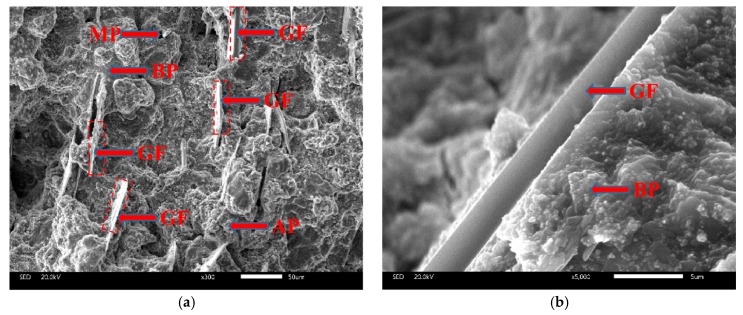
SEM images of the cross-section in GCBPCs: (**a**) 300×; (**b**) 5000×. Notes: AP (alumina particles), BP (binding phases), MP (micro-porous).

The typical SEM micrographs of different coating surfaces after curing are shown in Figure 6. The coatings of CBPCs consist of alumina distributed in the MAP binder. The binding phase is produced by the reaction between MAP and the surface of the particles during the curing process. As can be seen, the solidified binder fills the space between the particles. There were differences in the micrograph. The particles of the CBPC coating have an inhomogeneous morphology, the main component of which is Al_2_O_3_, while the particles spread more evenly in GCBPCs, which has a denser structure due to the new bonding phase. The result is consistent with the results of DSC. Furthermore, the GF particles on the surface and internal of the coating is shown in Figure 6 and Figure 7. The GF particles and the GCBPC maintain a good adhesion. On the other hand, the GF particles achieve a homogeneous and parallel dispersion on the surface and internal of the coatings.

### 3.3. Anti-Corrosion Performance

#### 3.3.1. Potentiodynamic Polarization

**Figure 8:**
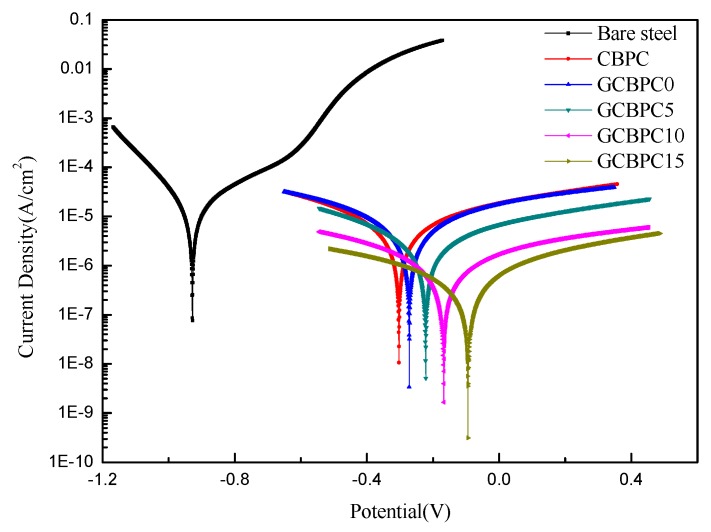
Potentiodynamic polarization curves of phosphate ceramic coatings.

The effect of the magnesia and the GF particles on the corrosion resistance of CBPCs samples was evaluated by using the polarization measurement in 3.5 wt.% NaCl solution. Figure 8 illustrates the potentiodynamic polarization data of the uncoated round steel, and the coated different phosphate ceramic samples. 

The corresponding electrochemical parameters are shown in Table 5, derived using the Tafel extrapolation method. The results show that the GCBPC0 has a higher E_corr_ and a lower i_corr_ compared to CBPC, which means the GCBPC0 has a better corrosion resistance than the CBPC. Meanwhile, with the increase of the GF in the GCBPCs, the value of E_corr_ increases, and the value of i_corr_ and corrosion rate decreases, indicating that the presence of GF can improve the anti-corrosion performance of the CBPCs [37]. In the GCBPC coating samples, the value of E_corr_ of GCBPC15 increases from −0.272 V of GCBPC0 to −0.094 V. Additionally, the value of i_corr_ and corrosion rate decreases from 7.396 × 10^−6^ A/cm^2^, 0.087 mm/a to 4.522 × 10^−7^ A/cm^2^, 0.005 mm/a, respectively. The reason for this may attributed to the most compact microstructure and the strongest resistance to electrolytes penetration being in GCBPC15. The effective inhibition *η* is calculated from Equation (4) [38].

(4) $$\eta =\left(1-\frac{i_{\mathrm{corr}}}{i_{\mathrm{corr},\mathrm{blank}}}\right)\times 100\%$$

where i_corr,blank_ is the etching current density of the uncoated sample, while i_corr_ represents the corrosion current density of the coated samples. As is well known, the higher the value of η, the better the anti-corrosion performance may be.

#### 3.3.2. Electrochemical Impedance Spectroscopy

**Figure 9:**
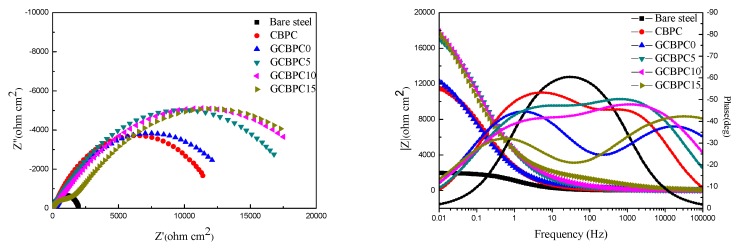
Nyquist and Bode plots of CBPCs.

The EIS method is an effective way to access the anticorrosion property of the CBPCs samples. The Nyquist and Bode plots of the CBPC coated samples immersed for 10 h in 3.5 wt.% NaCl solution are shown in Figure 9. EIS parameters (Table 6) from Nyquist and Bode plots were extracted by using an electrical equivalent circuit (R(RQ(RQ))) to model the experiment. R_s_, R_c_, CPE_c_, R_ct_ and CPE_ct_ represent resistance of solution, resistance of the CBPC coating, nonideal capacity of the CBPC coating, resistance of the charge transfer and nonideal capacity of the double layer, respectively.

According to EIS parameters from Table 6, the resistance of the solution (R_s_) remains stable, the addition of magnesia improves the anti-corrosion performance of CBPC to a certain extent, as the coating resistance (R_c_) and charge transfer resistance (R_ct_) increase. Meanwhile, the more GF added within the range of experimental dosage, the better anti-corrosion performance achieved. In addition, the GCBPC15 obtains the strongest anticorrosion property. According to the previous results (DSC, SEM and XRD), two main reasons can be noted for this phenomenon. Firstly, the presence of magnesia contributes to the formation of new phase and increases the compactness of GCBPCs to shield the round steels from the erosion of aggressive electrolyte. Secondly, the parallel distribution of GF particles on the surface and internal of the coating make the electrolyte diffusion path more tortuous. 

The comparison of the EIS parameters from different coatings between the previous literature and the results obtained in this research is shown in Table 7. As can be seen, the anti-corrosion property of the epoxy coating is significantly higher than other species of coatings due to the excellent protection against aggressive substances of the polymer matrix. For other types of coatings, GCBPC15 in this paper has better corrosion resistance performance than other coatings, which can mainly be attributed to the compactness of the matrix and the parallel distribution of GF particles.

### 3.4. Corrosion Protection Mechanism

As is widely known, the initial corrosion is commonly due to the penetration of sufficient H_2_O, O_2_, Cl^−^, or their combined effect on the steel [2,41]. The corrosion will cease to exist until the penetration is inhibited. For CBPCs, the substrate is protected by the compact coatings, which can retard the substrate corrosion under in cases with H_2_O, O_2_ and Cl^−^. The surface microstructures of the CBPC coatings are presented in Figure 7. Compared to GCBPC0, the GF particles are found in phosphate ceramics coatings, as shown in Figure 7 and Figure 8. The reason the GCBPC10 has better anticorrosion performance than GCBPC0 is that the new bonding phase and GF can hinder the permeation pathway of H_2_O, O_2_ and Cl^−^ and make the permeation more circuitous.

**Figure 10:**
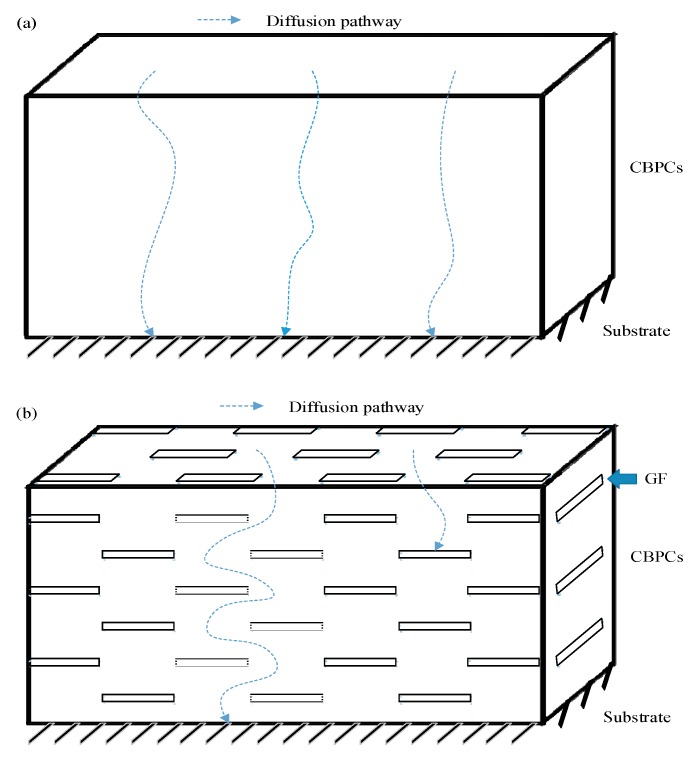
Schematic diagram of the anti-corrosion mechanism in (**a**) CBPCs and (**b**) GCBPCs.

These results indicate that GF-reinforced chemically bonded phosphate ceramic coatings are potentially superior compared to the neat chemically bonded phosphate ceramics in terms of anti-corrosion property. To clarify the anticorrosion performance, a model of GF reinforced chemically bonded phosphate ceramic coating was established. As shown in Figure 10, for CBPC or GCBPC0, aggressive substances such as H_2_O, O_2_ and Cl^−^ can be passed to the substrate through the diffusion route between the binding phase and the particle. However, the binding phase and the GF can hinder the aggressive substances permeation pathway and make the diffusion route more tortuous in GCBPCs, preventing H_2_O, O_2_ and Cl^−^ from engaging the substrate effectively.

## 4. Conclusions

The glass flake-reinforced chemically bonded phosphate ceramic coatings were prepared on round steel. The crystalline phase, curing behavior, micromorphology and electrochemical performance of the coating were studied. The main conclusions can be obtained as follows:

(1) With the addition of magnesia, a new bonding phase (Mg_3_(PO_4_)_2_) can be formed, which can help the GCBPCs obtain a more compact and denser structure. Meanwhile, The GF particles have a good adhesion with GCBPC and achieve a homogeneous and parallel dispersion on the surface and internal of the coatings.
(2) The effect of the magnesia and the GF additives on curing behavior is obvious; the heat of reaction of the phosphate ceramic materials increases, which emphasizes the higher crosslinking density in the phosphate ceramic materials.
(3) The phosphate ceramic coatings with the magnesia have a higher impedance value compared with the neat phosphate ceramic coating, while the highest impedance value is acquired with increase content of GF. It is found that GCBPC0 has a smaller particle size and a denser structure due to the new bonding phase compared with CBPC, and GF is distributed parallel on the surface and internal of the GCBPCs homogeneously. The corrosion mechanism is mainly contributed by the new bonding phase and GF, which can hinder the permeation pathway and make the permeation more circuitous.

## Figures and Tables

**Table 1:** Raw material of phosphate ceramic materials.

Name	Chemical Formula	Manufacturer
Monoaluminium phosphate	Al(H_2_PO_4_)_3_	-
Chromium trioxide	CrO_3_	Sinopharm Chemical Reagent Co., Ltd., Shanghai, China
Alumina	Al_2_O_3_	Aladdin Industrial Corporation Tech Co., Ltd., Shanghai, China
Magnesia	MgO	Sinopharm Chemical Reagent Co., Ltd., Shanghai, China
Glass flake (150 mesh)	SiO_2_	Hebei Huawei Glass Flake Co., Ltd., Langfang, China

**Table 2:** Mixture proportion of phosphate ceramic coating pastes.

Sample	MAP (g)	Powders (g)			H_2_O (g)
		Al_2_O_3_	MgO	GF	
CBPC	10.0	10.0	-	-	5.0
GCBPC0	10.0	9.5	0.5	0	5.0
GCBPC5	10.0	9.0	0.5	0.5	5.0
GCBPC10	10.0	8.5	0.5	1.0	5.0
GCBPC15	10.0	8.0	0.5	1.5	5.0

**Table 3:** Thickness of phosphate ceramic coatings after curing.

	Coatings				
	CBPC	GCBPC0	GCBPC5	GCBPC10	GCBPC15
Thickness (μm)	186	193	201	210	217
Standard deviation	0.3	0.2	0.2	0.3	0.2

**Table 4:** Curing parameters from DSC curves.

Sample	T_onset_ (°C)	T_p_ (°C)	T_endset_ (°C)	ΔH (J/g)
CBPC	209.5	228.0	245.5	−30.32
GCBPC0	118.9	126.9	153.9	−37.65
GCBPC5	117.8	126.3	153.3	−48.30
GCBPC10	115.4	125.4	153.4	−54.38
GCBPC15	113.7	125.2	153.2	−56.39

where T_onset_, T_p_, T_endset_ represents the temperature values of the onset, the peak and the endset, respectively, ΔH is the curing enthalpy.

**Table 5:** Curing parameters based on the potentiodynamic polarization curves.

Sample	Electrochemical Parameter			η (%)
	E_corr_ (V)	i_corr_ (A/cm^2^)	Corrosion Rate (mm/a)	
Bare Steel	−0.928	1.503 × 10^−5^	0.177	-
CBPC	−0.303	8.312 × 10^−6^	0.098	44.69
GCBPC0	−0.272	7.396 × 10^−6^	0.087	50.79
GCBPC5	−0.222	3.134 × 10^−6^	0.037	79.15
GCBPC10	−0.157	1.082 × 10^−6^	0.013	92.80
GCBPC15	−0.094	4.522 × 10^−7^	0.005	96.99

where E_corr_, i_corr_ represent the corrosion potential and corrosion current density, η is the effective inhibition, which is calculated from Equation (4).

**Table 6:** EIS parameters from Nyquist and Bode plots.

Sample	R_s_ (Ω·cm^2^)	R_c_ (Ω·cm^2^)	CPE_c_ (Ω·cm^2^)	N_c_	R_ct_ (Ω·cm^2^)	CPE_ct_ (F/cm^2^)	N_ct_
Bare Steel	9.46	-	-	-	1989	1.4327 × 10^−4^	0.75
CBPC	11.43	267	4.1701 × 10^−5^	0.69	12406	5.4811 × 10^−5^	0.67
GCBPC0	11.27	371	2.0892 × 10^−5^	0.57	14187	9.5405 × 10^−5^	0.64
GCBPC5	10.98	1128	3.1458 × 10^−5^	0.65	18370	3.6515 × 10^−5^	0.58
GCBPC10	10.90	1509	2.9823 × 10^−5^	0.61	20619	4.785 × 10^−5^	0.53
GCBPC15	10.88	1784	9.6835 × 10^−6^	0.54	20950	8.5690 × 10^−5^	0.57

where R_s_, R_c_, CPE_c_, R_ct_ and CPE_ct_ represent the resistance of the solution, resistance of the CBPC coating, the nonideal capacity of the CBPC coating, the resistance of charge transfer and the nonideal capacity of the double layer, respectively.

**Table 7:** Comparison of the EIS parameters from different coatings.

Species	R_s_ (Ω·cm^2^)	R_c_ (Ω·cm^2^)	CPE_c_ (Ω·cm^2^)	N_c_	R_ct_ (Ω·cm^2^)	CPE_ct_ (F/cm^2^)	N_ct_
CBPC	11.43	267	4.1701 × 10^−5^	0.69	12406	5.4811 × 10^−5^	0.67
GCBPC15	10.88	1784	9.6835 × 10^−6^	0.54	20950	8.5690 × 10^−5^	0.57
Epoxy [39]	100	28935	4.3417 × 10^−6^	0.75	44523	3.8667 × 10^−6^	0.22
Galvanized [40]	10	491.6	4.0417 × 10^−5^	0.80	2000	3.2378 × 10^−5^	0.66
GCBPC0-ZnO [24]	-	817.2	7.2100 × 10^−5^	0.64	911.7	3.8300 × 10^−5^	0.79

where R_s_, R_c_, CPE_c_, R_ct_ and CPE_ct_ represent the resistance of the solution, the resistance of the coating, the nonideal capacity of the coating, the resistance of the charge transfer and the nonideal capacity of the double layer, respectively.
